# Elevated TyG-BMI significantly increases the 1-year stroke recurrence risk in patients with acute ischemic stroke and hypertension

**DOI:** 10.3389/fendo.2025.1663393

**Published:** 2025-10-22

**Authors:** Yan Liu, Zhongzhong Liu, Qingli Lu, Pei Liu, Mi Zhang, Qiaoqiao Chang, Tong Liu, Linna Peng, Lanping Rao, Chao Sun, Guo Li, Shundao Cao, Xuemei Lin, Songdi Wu

**Affiliations:** ^1^ Department of Neurology, Xi’an No. 1 Hospital, The First Affiliated Hospital of Northwest University, Xi’an, China; ^2^ Xi’an Key Laboratory for Innovation and Translation of Neuroimmunological Diseases, Xi’an, China; ^3^ Department of Epidemiology and Biostatistics, School of Public Health of Xi’an Jiaotong University Health Science Center, Xi’an, China; ^4^ College of Life Science, Northwest University, Xi’an, China

**Keywords:** ischemic stroke, TyG-BMI, stroke recurrence, hypertension, sex differences

## Abstract

**Objectives:**

To investigate the association between triglyceride-glucose-body mass index (TyG-BMI) and the 1-year stroke recurrence risk in patients with acute ischemic stroke (AIS) and hypertension.

**Methods:**

In this retrospective, multicenter cohort study, multivariable Cox proportional hazards models were constructed, and curve fitting and subgroup analyses were performed to evaluate the aforementioned association. TyG-BMI was analyzed as a continuous variable and in quartiles (Q1–Q4). Sex-specific stratified analyses were performed to explore potential effect modifications.

**Results:**

Among 1,620 enrolled patients (39.6% women; mean age 65.2 ± 11.5 years), elevated TyG-BMI was significantly associated with increased 1-year stroke recurrence risk after adjusting for potential confounding factors (hazard ratio [HR]=1.06, 95% confidence interval [CI]: 1.01–1.11, P = 0.032). This association was particularly prominent in women (HR = 1.18, 95%CI: 1.07–1.29, P<0.001). Women in the Q3 and Q4 TyG-BMI groups had significantly higher 1-year stroke recurrence risks (Q3: HR=8.81 95%CI: 2.22–34.97, P = 0.002; Q4: HR=5.79 95%CI: 1.49–22.56, P = 0.011) compared with those in the Q1 group. No significant association was observed in men (HR = 1.01, 95%CI: 0.94–1.08, P = 0.830). Segmented linear regression and curve fitting revealed a significant sex-specific nonlinear relationship between TyG-BMI and 1-year stroke recurrence risk. For women when TyG-BMI was below 221.97, each unit increase was associated with a more pronounced increase in the risk of 1-year stroke recurrence compared to patients above this threshold (adjusted HR = 1.04, 95% CI 1.01–1.07, P = 0.005).

**Conclusion:**

Elevated TyG-BMI is independently associated with a higher risk of 1-year stroke recurrence in patients with AIS and hypertension. A significant association was identified only in women, specifically among those with a TyG-BMI below 221.97, below which each unit increase in TyG-BMI was associated with a significantly greater risk of 1-year stroke recurrence compared to those above this threshold.

## 1 Introduction

Stroke is the second leading cause of death globally and results in approximately 6.7 million deaths annually, accounting for over 116 million disability-adjusted life-years lost (1). Approximately 50% of patients with acute ischemic stroke (AIS) have comorbid hypertension (2), and this population faces a significantly higher risk of stroke recurrence compared to patients without hypertension (12–13% recurrence rate within 1 year) (3–5), posing a critical challenge for public health and clinical management. Despite the inclusion of antiplatelet, antihypertensive, and lipid-lowering therapies in secondary stroke prevention guidelines, the stroke recurrence risk among patients with hypertension remains persistently elevated, underscoring the limitations of conventional risk factor management.

In recent years, metabolic disorders have gained increasing attention as “silent killers” in the medical field. Insulin resistance (IR) participates in the pathological process of stroke through mechanisms, such as pro-inflammatory effects, endothelial dysfunction, and atherosclerosis (6–8). Multiple studies have substantiated that patients with IR face a significantly elevated risk of stroke recurrence (9, 10). Robust evidence confirms that the triglyceride-glucose index (TyG), calculated as the product of fasting plasma glucose (FPG) and triglyceride (TG) levels, serves as a simple, reproducible, and reliable indicator for assessing IR (11, 12). Numerous studies have validated the association between this index and stroke risk (13–16). Furthermore, body mass index (BMI) is often used as a standard for measuring obesity, and there has been a significant positive correlation between BMI and recurrent ischemic stroke recorded in various studies (17, 18). Recently, a novel index termed the triglyceride-glucose-BMI (TyG-BMI), calculated as the product of BMI and the TyG, has garnered significant attention. This composite metric captures concurrent variations in multiple clinical variables, including BMI, glycemic status, and lipid profiles, thereby reflecting dual metabolic abnormalities of IR and obesity (19). Previous studies have demonstrated that the TyG-BMI exhibits superior predictive performance compared to single-component indices (e.g., TyG and BMI) for cardiovascular diseases, diabetes risk, hypertension, and non-alcoholic fatty liver disease (20–23). A recent cohort study further elucidated this association, revealing that patients with elevated TyG-BMI indices and concomitant hypertension demonstrated a 3.49-fold increased risk of incident stroke compared to normotensive individuals with lower TyG-BMI (adjusted hazard ratio [HR] 3.49, 95% confidence interval [CI] 2.63–4.62). These findings underscore the clinical value of incorporating TyG-BMI assessment with conventional cardiovascular risk evaluation in developing multimorbidity prediction models (24).

Based on the known positive links between TyG, BMI, and stroke recurrence risk, alongside the established importance of IR in stroke development, we proposed that TyG-BMI might be associated with recurrent stroke risk. However, research on the association between TyG-BMI and the risk of stroke recurrence remains considerably limited, particularly within the critical subgroup of people with hypertension. To address this knowledge gap, we aimed to investigate the relationship between the TyG-BMI and 1-year stroke recurrence in patients with AIS and hypertension, utilizing longitudinal data from the Xi’an Stroke Registry Cohort.

## 2 Methods

### 2.1 Study design and study population

**Figure 1:**
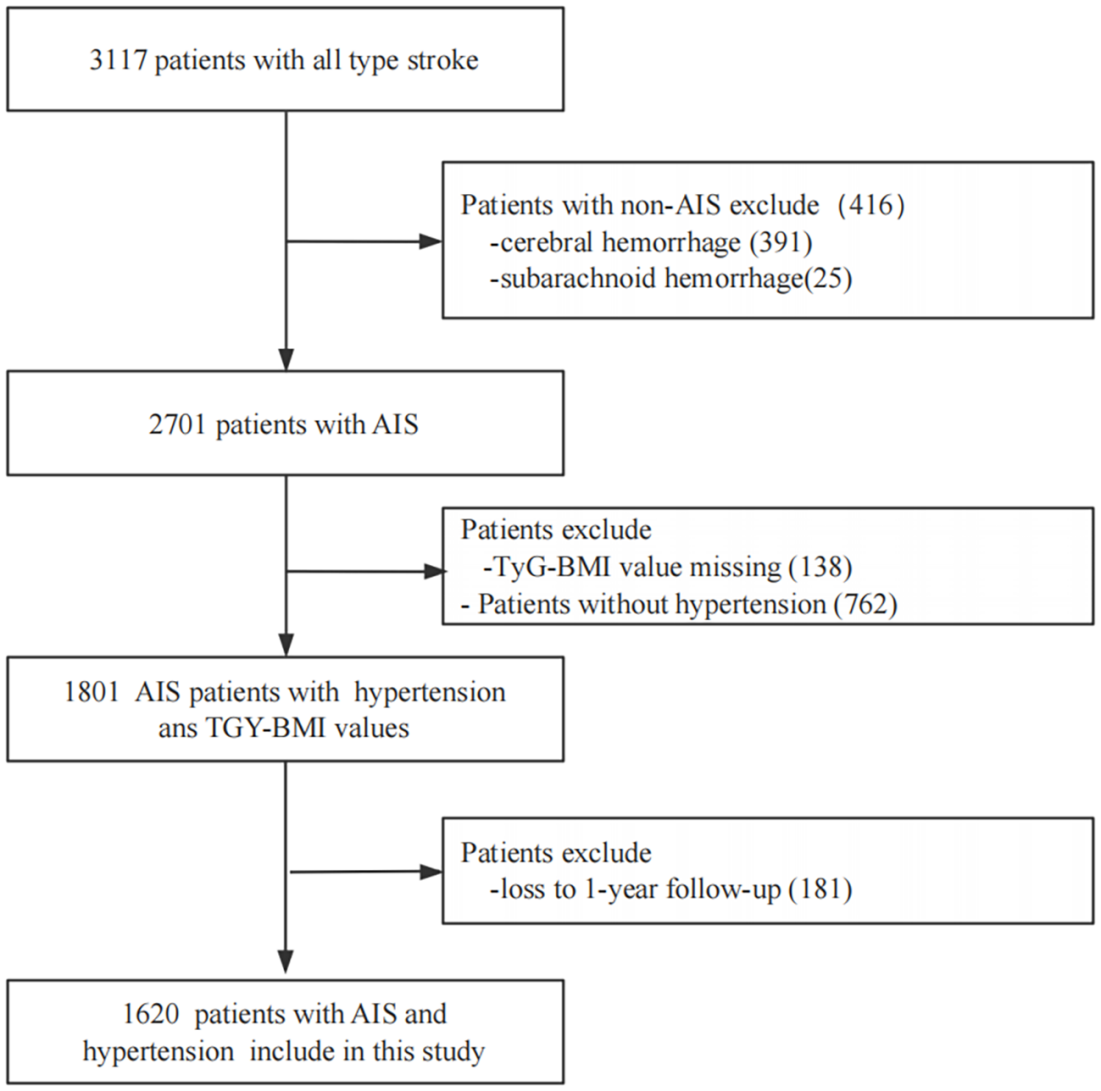
Screening flow charts for enrolled patients. AIS, acute ischemicstroke; TyG-BMI, triglyceride glucose-body mass index.

This was a multicenter, retrospective, observational cohort study. The study population comprised patients with stroke hospitalized between January and December 2015 at four tertiary Grade-A hospitals in Xi’an, China. At study initiation, 3,117 enrolled stroke patients received comprehensive clinical assessments and were followed longitudinally at 1, 3, 6, and 12 months following symptom onset. Inclusion criteria comprised (1): clinical diagnosis of AIS and hypertension; (2) brain computed tomography or cranial magnetic resonance findings meeting American Heart Association/American Stroke Association diagnostic criteria (25); (3) aged ≥ 18 years; and (4) symptom onset ≤ 7 days before study entry. Individuals were excluded for: (1) patients with cerebral hemorrhage or subarachnoid hemorrhage; (2) TyG-BMI values missing; (3) patients without hypertension; and (4) loss to 1-year follow-up. The screening workflow and study enrollment are illustrated in **Figure 1**. Consistent diagnostic criteria were applied across all participating hospitals.

### 2.2 Standard protocol approval and patient consent

This research was conducted in accordance with the principles of the Declaration of Helsinki. Ethical clearance was secured from the Academic Committee of Xi’an No.1 Hospital and the Ethics Committees of all collaborating institutions (Approval No. 2014 (5)). The trial was registered at ChiCTR-EOC-17012190. Written informed consent was provided by all participants prior to study enrollment.

### 2.3 Baseline data collection

This study utilized data from the Xi’an Stroke Registry. Baseline information was collected, including sociodemographic details (age, sex, educational level), medical history (smoking, drinking, pneumonia, peripheral vascular disease, prior stroke, diabetes mellitus, and atrial fibrillation), admission assessments (systolic blood pressure [SBP] on admission, diastolic blood pressure [DBP] on admission, heart rate, National Institutes of Health Stroke Scale [NIHSS] score on admission, walking within 48 h of admission), BMI, and key laboratory results (total cholesterol, TG, high-density lipoprotein [HDL] cholesterol, low-density lipoprotein [LDL] cholesterol, FPG, alanine aminotransferase, aspartate aminotransferase, alkaline phosphatase, homocysteine, serum creatinine, blood urea nitrogen, and uric acid levels; estimated glomerular filtration rate [eGFR]; international normalized ratio [INR]; leukocyte count; and platelet count). Definitions for medical history, risk factors, and study parameters were aligned with the China National Stroke Registry (CNSR) protocol (26). TyG-BMI was analyzed both as a continuous measure and categorized into quartiles (Q1–Q4). The participants were divided into quartiles based on their TyG-BMI index, using the following cutoff points: 190.20, 210.24, and 231.75. Thus, the quartiles (Q1-Q4) were defined as Q1: <190.20, Q2: 190.20–210.24, Q3: 210.24–231.75, and Q4: ≥231.76.

### 2.4 Measurements and outcomes

AIS in this research is defined as a group of disorders involving compromised cerebral perfusion due to diverse causes, leading to ischemic-hypoxic brain tissue necrosis and consequent neurological deficits. This category encompasses acute cerebral infarction and transient ischemic attack, with diagnoses established per World Health Organization criteriab (27). Fasting venous blood samples were collected within 24 hours after admission for laboratory testing. All serum samples were analyzed within 2 hours of collection. Each center performed routine laboratory tests using an automated biochemical analyzer, including FPG and blood lipid profile. FPG and fasting TG levels were measured using the hexokinase method and enzymatic colorimetry, respectively. TyG-BMI levels were calculated as Ln [FPG (mg/dL) × TG (mg/dL)/2] × BMI (kg/m^2^) (28). Smoking status was defined as the regular consumption of ≥ 1 cigarette per day for at least 6 months prior to stroke onset. Smoking cessation was defined as meeting the historical smoking criteria but having abstained for at least 6 months before the stroke. Alcohol consumption was defined as intake of ≥1 standard drink weekly; a standard drink was equivalent to 45 mL of spirits, 360 mL of beer, or 120 mL of wine. Pneumonia diagnosis within the first week post-stroke (excluding mechanically ventilated patients) required clinical manifestations (e.g., fever, purulent sputum, cough), supportive chest X-ray findings, and laboratory abnormalities (such as altered leukocyte counts) (29). The primary endpoint of stroke recurrence in this investigation was defined as the occurrence of a new cerebrovascular event (including cerebral infarction, intracerebral hemorrhage, or subarachnoid hemorrhage) occurring within the 1-year follow-up. Endpoint adjudication was carried out by a dedicated committee comprising 4 to 5 stroke specialists from each participating center.

### 2.5 Follow-up

All patients included in this retrospective study underwent routine follow-up at 1, 3, 6, and 12 months after AIS diagnosis. Follow-up was conducted via telephone or in-person visits by trained research coordinators to ensure that follow-up times were not deviated by more than 5 days and that the date of recurrent stroke was accurately recorded. Participants were considered lost to follow-up if they voluntarily withdrew from the study or were unable to be reached despite three contact attempts per day for five consecutive working days.

### 2.6 Statistical analyses

Normally distributed continuous variables are presented as mean ± standard deviation. Categorical variables are expressed as frequencies (percentages). Non-normally distributed continuous variables are reported as median (interquartile range). For group comparisons, one-way analysis of variance, chi-square tests, or the Kruskal-Wallis test were employed based on data distribution characteristics. Fisher’s exact test was employed when expected cell counts were below 10. The covariate selection in our study was based on three main criteria: First, collinearity screening: we conducted a collinearity check for the independent variables using the variance inflation factor (VIF), where VIF = 1/(1 - R²). A higher VIF indicates greater collinearity. Variables with a VIF ≥ 10 were excluded from the models. Second, the P-value and change in regression coefficients: covariates were selected based on their regression coefficients with P-values < 0.10, or if the inclusion of the covariate in the baseline model led to a change in the hazard ratio coefficients of >10%. Third, clinical significance and literature support: covariates were also chosen based on previous literature and clinical relevance, considering the practical context of our study. The study includes two adjustment models. Model I is a minimal adjustment, which adjusts for routine and generally necessary variables (including age, education, smoking, and alcohol consumption). Model II involves selecting potential variables that may influence the association between TyG-BMI and stroke recurrence based on covariate screening criteria, and incorporating them into a multivariate regression model to exclude potential confounders, thus determining the independent relationship between TyG-BMI and stroke recurrence. Multivariable Cox proportional hazards regression models were constructed to evaluate the association between TyG-BMI and 1-year stroke recurrence in patients with AIS and hypertension. Receiver operating characteristic (ROC) curves were used to evaluate the predictive value of TyG-BMI and its components for 1-year stroke recurrence. In addition, smoothed curve fitting and a two-piecewise linear regression model were employed to investigate the threshold effect of TyG-BMI on the risk of 1-year stroke recurrence. Likelihood ratio tests were conducted to compare the goodness-of-fit between linear and nonlinear models. Subgroup analyses were performed to investigate potential interaction effects across stratified variables. Statistical significance was defined as a two-sided P-value < 0.05. All analyses were executed using R statistical software (version 4.2.0; R Foundation for Statistical Computing, Vienna, Austria) and EmpowerStats (version 4.2; X&Y Solutions, Inc., Boston, MA, USA).

## 3 Results

### 3.1 Baseline characteristics

After 1 year of follow-up, 181 participants either withdrew or were lost to contact, resulting in a final analytic cohort of 1,620 individuals (978 men and 642 women) with a mean age of 65.2 ± 11.5 years. The baseline demographic, clinical, and laboratory profiles were stratified and compared according to TyG-BMI quartile (Q1–Q4), as detailed in **Table 1**. There were significant differences in age; educational level; BMI; DBP on admission; heart rate; presence of co-morbidities, including diabetes mellitus and atrial fibrillation; NIHSS score; blood parameters, including total cholesterol, TG, HDL cholesterol, LDL cholesterol, FPG, TyG, alanine aminotransferase, eGFR, uric acid, leukocyte count, INR; Pharmacotherapy information, including antidiabetic drug therapy and lipid-lowering drug therapy among different TyG-BMI levels (P < 0.05). In addition, we further divided the patients into two groups based on 1-year stroke recurrence (with stroke recurrence and without stroke recurrence) and described and analyzed their baseline clinical characteristics (**Supplementary Table 1**). The results showed that age, heart rate, NIHSS score on admission, walking within 48 hours of admission, pneumonia, prior stroke, atrial fibrillation, FPG, alkaline phosphatase, homocysteine, eGFR, blood urea nitrogen, leukocyte count, and antithrombotic drug therapy showed significant differences between the two groups.

**Table 1:** Baseline characteristics by TyG-BMI level quartiles (Q1-Q4) in patients with AIS and hypertension.

Variables	TyG-BMI level quartiles					P-value
	Total, (n = 1620)	Q1, n=405	Q2, n=405	Q3, n=405	Q4, n=405	
Age, years	65.2 ± 11.5	68.5 ± 11.8	66.0 ± 10.7	64.5 ± 11.2	61.9 ± 11.3	< 0.001
Sex						0.283
men	978 (60.4)	244 (60.2)	252 (62.2)	253 (62.5)	229 (56.5)	
women	642 (39.6)	161 (39.8)	153 (37.8)	152 (37.5)	176 (43.5)	
Educational level						0.002
elementary or below	764 (47.2)	220 (54.3)	198 (48.9)	164 (40.5)	182 (44.9)	
middle school	333 (20.6)	72 (17.8)	91 (22.5)	86 (21.2)	84 (20.7)	
high school or above	523 (32.3)	113 (27.9)	116 (28.6)	155 (38.3)	139 (34.3)	
Smoking, n (%)						0.896
Never smoking	950 (58.6)	233 (57.5)	242 (59.8)	234 (57.8)	241 (59.5)	
smoking cessation	322 (19.9)	81 (20)	83 (20.5)	85 (21)	73 (18)	
current smoking	348 (21.5)	91 (22.5)	80 (19.8)	86 (21.2)	91 (22.5)	
Drinking	369 (22.8)	77 (19)	94 (23.2)	96 (23.7)	102 (25.2)	0.184
Time-to hospital admission	42.4 ± 45.2	39.5 ± 43.9	44.8 ± 47.1	41.9 ± 45.7	43.5 ± 44.1	0.386
BMI, kg/m2	24.1 ± 3.6	20.6 ± 2.1	23.3 ± 1.3	24.7 ± 1.4	27.9 ± 3.9	< 0.001
SBP on admission (mmHg)	150.4 ± 21.6	149.0 ± 23.0	151.6 ± 21.5	150.2 ± 21.1	150.8 ± 20.9	0.377
DBP on admission (mmHg)	87.6 ± 12.7	85.8 ± 12.3	88.2 ± 12.8	87.7 ± 12.5	88.8 ± 13.0	0.005
Heart rate (times per minute)	74.9 ± 10.5	74.2 ± 10.9	74.0 ± 9.8	74.9 ± 10.0	76.4 ± 11.0	0.005
NIHSS score on admission	4.0 (2.0, 6.0)	4.0 (2.0, 7.0)	4.0 (2.0, 6.0)	4.0 (1.0, 5.0)	4.0 (2.0, 6.0)	0.013
Walking within 48 hours of admission	959 (64.0)	232 (61.5)	238 (64.2)	242 (65.2)	247 (65.2)	0.691
Pneumonia, n (%)	85 (5.2)	27 (6.7)	16 (4)	21 (5.2)	21 (5.2)	0.389
Past medical history						
Peripheral vascular history	51 (3.1)	16 (4)	13 (3.2)	11 (2.7)	11 (2.7)	0.716
Prior stroke, n (%)	512 (31.6)	134 (33.1)	132 (32.6)	126 (31.1)	120 (29.6)	0.712
Diabetes mellitus, n (%)	432 (26.7)	53 (13.1)	87 (21.5)	120 (29.6)	172 (42.5)	< 0.001
Atrial fibrillation, n (%)	107 (6.6)	42 (10.4)	22 (5.4)	26 (6.4)	17 (4.2)	0.003
Total cholesterol (mmol/L)	4.4 ± 1.1	4.1 ± 1.0	4.4 ± 1.0	4.5 ± 1.0	4.8 ± 1.3	< 0.001
TG (mgldL)	156.0 ± 131.9	97.0 ± 48.3	125.9 ± 58.8	156.9 ± 76.5	244.1 ± 214.2	< 0.001
HDL-cholesterol (mmol/L)	1.1 ± 0.3	1.2 ± 0.3	1.1 ± 0.3	1.1 ± 0.3	1.1 ± 0.3	< 0.001
LDL-cholesterol (mmol/L)	2.6 ± 0.9	2.4 ± 0.8	2.6 ± 0.8	2.7 ± 0.8	2.9 ± 1.0	< 0.001
FPG (mgldL)	109.4 ± 43.5	94.7 ± 29.2	101.3 ± 29.7	109.7 ± 39.3	132.1 ± 59.3	< 0.001
TyG	8.8 ± 0.7	8.3 ± 0.5	8.6 ± 0.5	8.9 ± 0.5	9.4 ± 0.7	< 0.001
Alanine aminotransferase (U/L)	24.2 ± 21.8	20.4 ± 15.2	23.3 ± 24.9	25.3 ± 26.7	27.7 ± 17.7	< 0.001
Aspartate aminotransferase (U/L)	24.8 ± 17.9	23.5 ± 10.3	24.9 ± 22.3	24.7 ± 16.8	26.0 ± 19.8	0.294
Alkaline phosphatase (U/L)	79.6 ± 27.7	78.1 ± 28.0	80.5 ± 33.7	79.9 ± 22.8	79.8 ± 25.3	0.672
Homocysteine (µmol/mL)	21.7 ± 14.8	22.4 ± 15.9	22.1 ± 14.3	21.4 ± 14.9	20.8 ± 13.8	0.584
Serum creatinine (µmol/L)	77.1 ± 38.9	74.5 ± 34.7	77.2 ± 23.1	80.3 ± 52.8	76.1 ± 39.2	0.191
eGFR (mL/min/1.73m^2^)	74.7 ± 17.4	71.8 ± 17.9	75.3 ± 15.6	74.8 ± 17.4	76.8 ± 18.0	< 0.001
Blood urea nitrogen (mmol/L)	5.2 ± 1.9	5.4 ± 2.3	5.2 ± 1.7	5.1 ± 2.0	5.2 ± 1.8	0.083
INR	1.0 ± 0.2	1.1 ± 0.3	1.0 ± 0.2	1.0 ± 0.1	1.0 ± 0.2	< 0.001
Uric acid (µmol/L)	292.0 ± 98.2	272.3 ± 92.1	292.2 ± 94.3	301.0 ± 99.6	301.9 ± 103.6	< 0.001
Leukocyte count (×10^9^/L)	7.0 ± 2.5	6.7 ± 2.7	6.9 ± 2.3	7.0 ± 2.3	7.3 ± 2.6	0.014
Platelet count (×10^9^/L)	190.9 ± 60.3	188.9 ± 60.4	190.5 ± 62.3	189.3 ± 58.8	195.0 ± 59.6	0.466
Antithrombotic drug therapy, n (%)	1449 (89.4)	369 (91.1)	356 (87.9)	365 (90.1)	359 (88.6)	0.442
Antihypertensive drug therapy, n (%)	1173 (72.4)	282 (69.6)	289 (71.4)	294 (72.6)	308 (76)	0.214
Antidiabetic drug therapy, n (%)	346 (21.4)	39 (9.6)	65 (16)	98 (24.2)	144 (35.6)	< 0.001
Lipid-lowering drug therapy, n (%)	593 (36.6)	105 (25.9)	114 (28.1)	165 (40.7)	209 (51.6)	< 0.001
Anticoagulant drug therapy, n (%)	35 (2.2)	15 (3.7)	6 (1.5)	8 (2)	6 (1.5)	0.094

data presented are mean ± SD, median (Q1–Q3), or N (%).

BMI, body mass index; SBP, systolic blood pressure; DBP, diastolic blood pressure; NIHSS, national institutes of health stroke scale; TG, triglycerides; FPG, fasting plasma glucose; TyG, triglyceride glucose; HDL, high-density lipoprotein; LDL, low-density lipoprotein; eGFR, estimated glomerular filtration rate, INR, International Normalized Ratio. Q, quartilesQ1: <190.20, Q2:190.21-210.24, Q3: 210.25-231.75,Q4: ≥231.76.

### 3.2 Association between TyG-BMI levels and the 1-year stroke recurrence risk

Considering TyG-BMI as a continuous variable, multivariable Cox regression analysis, adjusted for confounders in model II, revealed that each 10-unit increase in TyG-BMI was associated with a 6% higher risk of 1-year stroke recurrence (HR = 1.06, 95% CI 1.01–1.11, P = 0.032; **Table 2**). Quartile analysis revealed significantly increased risks in Q3 (HR = 2.45, 95% CI 1.30–4.62, P = 0.006) and Q4 (HR = 2.27, 95% CI 1.16–4.46, P = 0.017) vs Q1 in model II. A statistically significant increasing trend in stroke recurrence risk was observed across the TyG-BMI quartiles (Q1 to Q4, P = 0.006; **Table 2**). In addition, we conducted a preliminary univariate analysis to explore the predictive value of TyG-BMI and its individual components (including FPG, TG, and BMI) for 1-year stroke recurrence in patients with AIS and hypertension. The results showed that TyG-BMI (C-statistics = 0.65), BMI (C-statistics = 0.54), TG (C-statistics = 0.47), and FPG (C-statistics = 0.56), suggesting that the predictive value of TyG-BMI was higher than that of its individual components (**Supplementary Figure 1**).

**Table 2:** Cox regression analysis of TyG-BMI levels and the 1-year stroke recurrence risk in patients with AIS and hypertension.

Variable	N.total	N.event %	Crude model HR (95% CI)	P value	Adjusted mode I HR(95%CI)	P value	Adjusted mode II HR(95%CI)	P value
TyG-BMI per 10 unit increase	1620	89 (5.5)	1.02 (0.97~1.07)	0.464	1.05 (1.00~1.10)	0.032	1.06 (1.01~1.11)	0.032
TyG-BMI quartile								
Q1	405	17 (4.2)	Ref.		Ref.		Ref.	
Q2	405	19 (4.7)	1.12 (0.58~2.16)	0.727	1.30 (0.67~2.52)	0.431	1.54 (0.78~3.05)	0.214
Q3	405	29 (7.2)	1.72 (0.95~3.14)	0.075	2.29 (1.24~4.22)	0.008	2.45 (1.30~4.62)	0.006
Q4	405	24 (5.9)	1.41 (0.76~2.63)	0.276	2.17 (1.15~4.12)	0.018	2.27 (1.16~4.46)	0.017
Trend test				0.141		0.004		0.006

Crude model adjust for: None; Adjustmodel I adjust for: age; educational level; smoking; drinking; Adjust model II adjust for: age; educational level; smoking; drinking; time-to hospital admission, prior stroke, NIHSS score on admission, pneumonia, alkaline phosphatase, eGFR, leukocyte count, diabetes mellitus, atrial fibrillation, antithrombotic drug therapy, antihypertensive drug therapy, antidiabetic drug therapy, lipid-lowering drug therapy, and anticoagulant drug therapy.

### 3.3 Subgroup analyses

**Figure 2:**
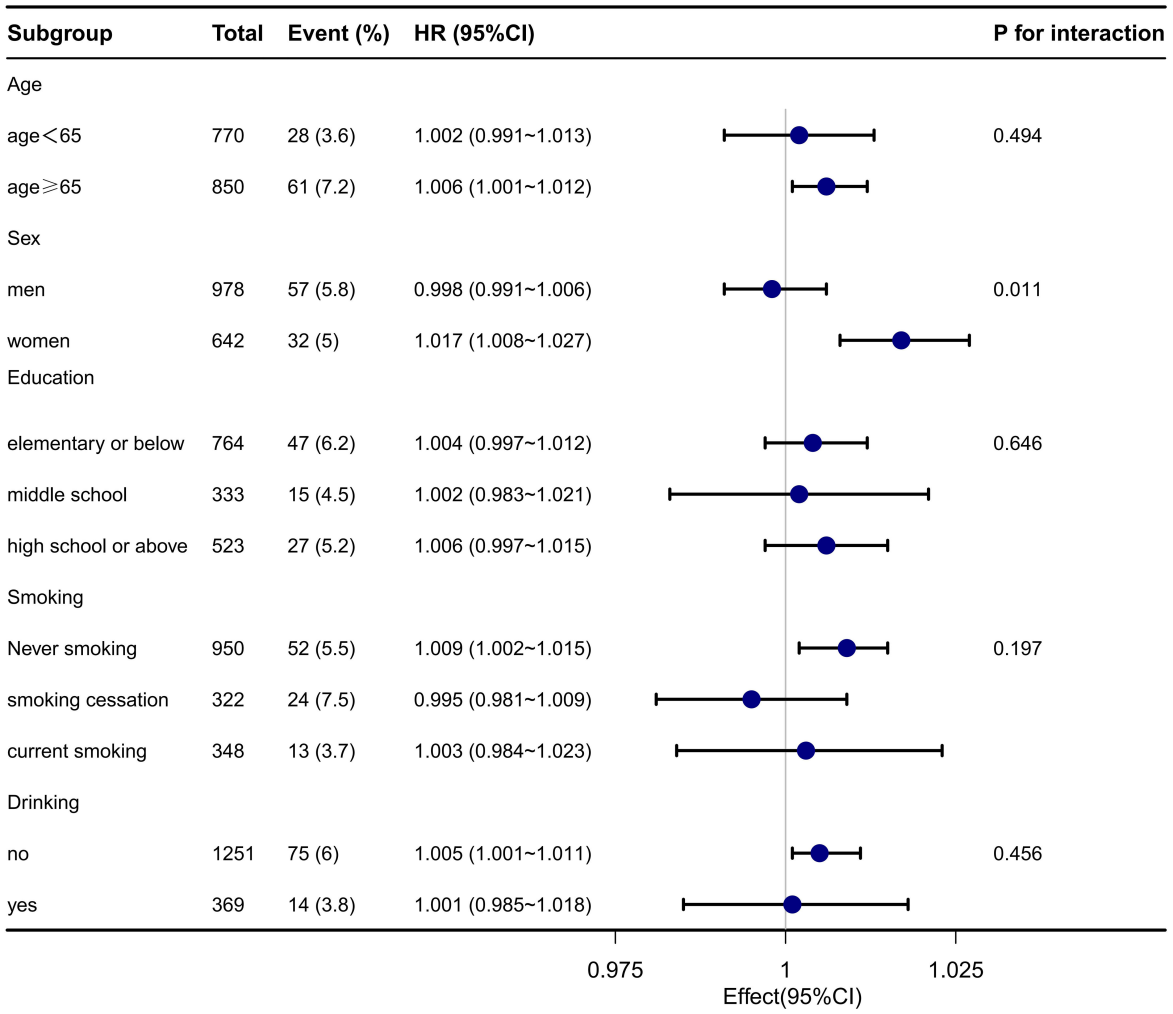
Subgroup analysis of TyG-BMI and the 1-year stroke recurrence risk in patients with AIS and hypertension.

Stratified and interaction analyses were conducted to assess the association between elevated TyG-BMI and the 1-year stroke recurrence risk across various clinically relevant subgroups in patients with AIS and hypertension. The results are presented in **Figure 2**. Significant interactions were found for sex (P for interaction = 0.011), suggesting that the impact of TyG-BMI on stroke recurrence risk may differ across this subgroup. No significant interactions were detected for age, educational level, drinking, and smoking status (all P > 0.05).

### 3.4 Association between TyG-BMI in different sexes and the 1-year stroke recurrence risk

In patients with AIS and hypertension, Cox regression stratified by sex revealed a significant divergence in TyG-BMI association with 1-year stroke recurrence risk (**Table 3**). Among men (n=978), neither the continuous TyG-BMI (per 10-unit increase: adjusted HR = 1.01, 95% CI 0.94–1.08, P = 0.830) nor the quartile-based analysis (Q4 vs. Q1: adjusted HR = 1.72, 95% CI 0.70–4.22, P = 0.233; P for trend = 0.16) demonstrated statistical significance after full adjustment for covariates (age, lifestyle, comorbidities, and clinical indices). Conversely, in women (n=642), each 10-unit TyG-BMI increase markedly elevated the risk of 1-year stroke recurrence (adjusted HR = 1.18, 95% CI:1.07–1.29, P < 0.001), with quartile analysis demonstrating a robust dose-response effect: Q3 (HR = 8.81, 95% CI 2.22–34.79, P = 0.002) and Q4 (HR = 5.79, 95% CI 1.49–22.56, P = 0.011) vs Q1, alongside a significant trend (P for trend = 0.002).

**Table 3:** Cox regression analysis of TyG-BMI levels of different sexes and the 1-year stroke recurrence risk in patients with AIS and hypertension.

Stratified analysis by sex	N.total	N.event_%	Crude model HR (95% CI)	P value	Adjusted mode I HR(95%CI)	P value	Adjusted mode II HR(95%CI)	P value
Men								
TyG-BMI per 10 unit increase	978	57 (5.8)	0.97 (0.91~1.05)	0.470	1.02 (0.95~1.09)	0.648	1.01 (0.94~1.08)	0.830
TyG-BMI quartile								
Q1	244	13 (5.3)	Ref.		Ref.		Ref.	
Q2	252	14 (5.6)	1.06 (0.5~2.25)	0.887	1.21 (0.57~2.59)	0.616	1.48 (0.68~3.26)	0.329
Q3	253	18 (7.1)	1.36 (0.67~2.78)	0.396	1.89 (0.91~3.94)	0.089	1.83 (0.84~3.93)	0.124
Q4	229	12 (5.2)	0.98 (0.45~2.15)	0.96	1.58 (0.7~3.57)	0.269	1.72 (0.70~4.22)	0.233
Trend.test				0.832		0.138		0.160
Women								
TyG-BMI per 10 unit increase	642	32 (5)	1.11 (1.02~1.21)	0.019	1.12 (1.04~1.2)	0.002	1.18 (1.07~1.29)	<0.001
TyG-BMI quartile								
Q1	161	4 (2.5)	Ref.		Ref.		Ref.	
Q2	153	5 (3.3)	1.31 (0.35~4.87)	0.689	1.55 (0.42~5.83)	0.512	1.53 (0.31~7.56)	0.605
Q3	152	11 (7.2)	2.91 (0.93~9.14)	0.067	3.82 (1.2~12.14)	0.023	8.81 (2.22~34.97)	0.002
Q4	176	12 (6.8)	2.76 (0.89~8.56)	0.079	3.9 (1.24~12.34)	0.02	5.79 (1.49~22.56)	0.011
Trend.test				0.033		0.006		0.002

Crude model adjust for: None; Adjustmodel I adjust for: age; educational level; smoking; drinking; Adjust model II adjust for: age; educational level; smoking; drinking, time-to hospital admission, prior stroke, NIHSS score on admission, pneumonia, alkaline phosphatase, eGFR, leukocyte count, diabetes mellitus, atrial fibrillation, antithrombotic drug therapy, antihypertensive drug therapy, antidiabetic drug therapy, lipid-lowering drug therapy, and anticoagulant drug therapy.

### 3.5 Threshold Analysis of TyG-BMI levels and the 1-year stroke recurrence risk by sex

**Figure 3:**
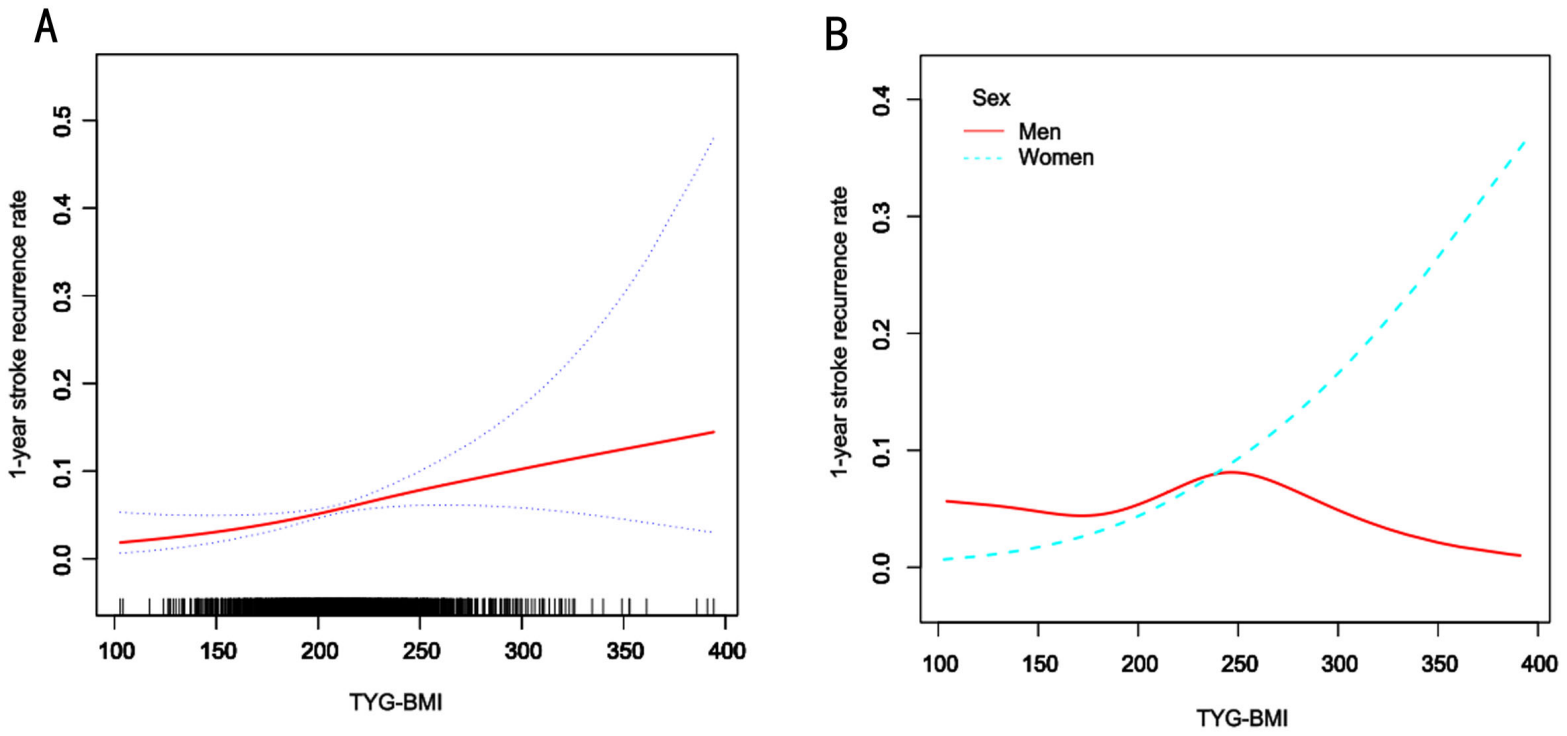
Smooth curve fitting of TyG-BMI to the risk of 1-year risk of stroke recurrence. A, Smooth curve fitting analysis of the relationship between TyG-BMI and 1-year stroke recurrence risk in all patients and the red line represents the overall trend, the blue dashed line indicates the confidence interval, and the black short lines on the horizontal axis represent the TyG-BMI distribution density. B, Smooth curve fitting analysis stratified by sex. The blue line represents women, and the red line represents men.

Piecewise Cox regression confirmed significant sex-specific nonlinearity in the association between TyG-BMI and the 1-year stroke recurrence risk among patients with AIS and hypertension (likelihood ratio test: P < 0.05 for all; **Table 4**). Women exhibited a critical inflection point at TyG-BMI = 221.97, below this threshold, for each one-unit increase in TyG-BMI, the risk of 1-year stroke recurrence in women patients increased by 4% (HR = 1.04, 95% CI 1.01–1.07, P = 0.005), whereas above it, each unit increase in TyG-BMI was not significantly associated with the risk of 1-year stroke recurrence (HR = 1.00, 95% CI 0.99–1.02, P = 0.678). In contrast, men showed no statistically significant associations across TyG-BMI levels despite an inflection point at TyG-BMI = 251.5 (both segmental P > 0.05). The overall cohort inflection point was TyG-BMI = 231.45, below this threshold, for each one-unit increase in TyG-BMI, the risk of 1-year stroke recurrence increased by 1% (HR = 1.01, 95% CI 1.00–1.02, P = 0.007), whereas above it, each unit increase in TyG-BMI was not significantly associated with the risk of 1-year stroke recurrence (HR = 1.00, 95% CI 0.99–1.01, P = 0.638). (adjusted for demographics, comorbidities, and clinical indices). Smooth curve fitting further confirmed a sex-divergent nonlinear relationship between TyG-BMI and the risk of 1-year stroke recurrence (**Figure 3AB**). In the overall population, recurrence risk increased progressively with higher TyG-BMI levels. Women exhibited a distinct J-shaped curve, whereas men demonstrated a flat association curve across TyG-BMI levels (**Figure 3B**).

**Table 4:** Association between TyG-BMI levels and the 1-year stroke recurrence risk in patients with AIS and hypertension based on sex using Piecewise Cox Regression analysis.

Threshold effect analysis	Sex				Total	
	Men		Women			
TyG-BMI levels	HR (95% CI)	P value	HR (95% CI)	P value	HR (95% CI)	P value
One-line linear regression model	1.00 (0.99, 1.01)	0.830	1.02 (1.01, 1.03)	<0.0001	1.01 (1.00, 1.01)	0.032
Two-piecewise linear regression model						
Inflection point (K)	251.5		221.97		231.45	
< K slope	1.01 (1.00, 1.02)	0.121	1.04 (1.01, 1.07)	0.005	1.01 (1.00, 1.02)	0.007
> K slope	0.96 (0.90, 1.02)	0.175	1.00 (0.99, 1.02)	0.678	1.00 (0.99, 1.01)	0.638
Likelihood ratio test		0.036		0.046		0.044

Adjust model adjust for: age; educational level; smoking; drinking; time-to hospital admission, prior stroke, NIHSS score on admission, pneumonia, alkaline phosphatase, eGFR, leukocyte count, diabetes mellitus, atrial fibrillation, antithrombotic drug therapy, antihypertensive drug therapy, antidiabetic drug therapy, lipid-lowering drug therapy, and anticoagulant drug therapy.

## 4 Discussion

Findings from this multicenter retrospective cohort study, drawing on the Xi’an Stroke Registry, indicated that higher TyG-BMI levels were significantly linked to an elevated 1-year stroke recurrence risk in patients with AIS and hypertension, particularly among women. Sex-stratified analysis, after adjusting for potential confounding factors and categorizing by TyG-BMI quartiles, demonstrated that women in the Q3 and Q4 groups had a significantly higher risk of 1-year stroke recurrence compared to those in Q1. A dose-dependent relationship was observed. Specifically, women with a TyG-BMI < 221.97 exhibited a significantly elevated 1-year stroke recurrence risk. In contrast, no significant association was observed in men. These findings underscore the need for clinicians to closely monitor TyG-BMI levels in patients with AIS and hypertension, particularly among women.

Ma et al. analyzed data from 6,922 individuals aged ≥ 45 years in the China Health and Retirement Longitudinal Study and validated the clinical utility of TyG-BMI in stroke primary prevention, demonstrating a 3.49-fold elevated stroke risk in hypertensive patients with high TyG-BMI versus their normotensive counterparts with lower TyG-BMI (adjusted HR = 3.49, 95% CI 2.63–4.62) (24). In the quartile-based analysis of TyG-BMI, they observed that the relationship between TyG-BMI and stroke risk was not significant in the hypertensive population, with an HR of 1.02 for the Q4 group (95% CI: 0.56–1.87, P > 0.05). This is not consistent with the results of the present multicenter study, where we found that elevated TyG-BMI was significantly associated with an increased 1-year stroke recurrence risk in patients with AIS and hypertension. The observed discrepancies in research findings may be attributed to factors such as composition of the patient population, sample size, research methodologies, and geographic distribution. Collectively, these stroke-related findings demonstrate a robust association between TyG-BMI and stroke incidence and progression. However, no prior studies have specifically investigated this association in patients with AIS and hypertension. To address this gap and examine potential causal links between TyG-BMI and stroke recurrence in this high-risk population, we conducted a retrospective cohort study of 1,620 patients with AIS and hypertension.

Yu et al. demonstrated that elevated TyG-BMI is associated with reduced stroke severity and improved clinical outcomes (30). In contrast, our study revealed that elevated TyG-BMI is significantly associated with increased 1-year stroke recurrence risk in patients with AIS and comorbid hypertension, particularly among women. Beyond variations in study designs and result patterns, their study utilized population-level data from Northeast China, whereas our analysis employed regional data from Northwest China, thus providing region-specific implications for tailored prevention strategies. However, prior research has predominantly focused on the associations between TyG-BMI and stroke prognosis (e.g., functional outcomes and mortality), investigations linking TyG-BMI to stroke recurrence remain strikingly underexplored.

Subgroup analyses from prior studies have indicated that individuals with elevated TyG-BMI and comorbid hypertension exhibit the highest stroke risk following adjustment for demographic characteristics, lifestyle factors, anthropometric measures, and chronic disease history. Notably, this association was particularly pronounced in women, demonstrating a significantly elevated incident stroke risk (HR = 2.62, 95% CI: 1.64–4.21) (24). Similarly, sex-stratified analyses in our cohort revealed a pronounced increase in recurrence risk among women with higher TyG-BMI quartiles (Q3: HR 11.64; Q4: HR 8.57), whereas no association was observed in men after multivariable adjustment.

Prior studies have demonstrated a nonlinear association between TyG-BMI and stroke risk, identifying a statistically significant threshold effect at TyG-BMI = 174.63 (31). Below this threshold, each 10-unit increment in TyG-BMI was associated with a steeper risk increment (HR = 1.144, 95% CI: 1.044–1.253). Conversely, above 174.63, the risk impact attenuated substantially (per 10-unit increase: HR = 1.038, 95% CI: 1.016–1.061). Consistent with our findings, this observation confirms a nonlinear association of TyG-BMI with 1-year stroke recurrence risk. Our study identified a statistically significant threshold effect for TyG-BMI at 231.45 in the overall AIS cohort. Below this threshold, each 1-unit increase in TyG-BMI was associated with a more pronounced increase in risk (HR = 1.01, 95% CI 1.00–1.02). Conversely, above 231.45, the impact on risk was substantially attenuated (per 1-unit increase: HR = 1.00, 95% CI 0.99–1.01). To further explain the details observed in the threshold saturation effect, we divided the patients into two groups: TyG-BMI < 231.45 and TyG-BMI ≥ 231.45, and compared their clinical characteristics. The results revealed that the TyG-BMI < 231.45 group had a higher proportion of older patients, a higher incidence of atrial fibrillation, and a lower proportion of patients receiving antidiabetic drug therapy and lipid-lowering drug therapy. We found that the risk of recurrent stroke at 1 year in the TyG-BMI < 231.45 group might be associated with these clinical characteristics. Clinicians should consider these factors when providing targeted interventions, which may effectively reduce the risk of recurrent stroke at 1 year (**Supplementary Table 2**). Unlike previous investigations, our subgroup analyses revealed a significant sex-specific threshold for TyG-BMI. Specifically, the piecewise regression analysis revealed that among females with a TyG-BMI below 221.97, each unit increase in TyG-BMI was associated with a significantly greater increase in the risk of 1-year stroke recurrence compared to those above this threshold (adjusted HR = 1.04, 95% CI 1.01–1.07, P = 0.005). These findings indicate the need for sex-tailored metabolic management strategies and highlight TyG-BMI as a promising prognostic indicator.

The mechanism by which elevated TyG-BMI levels are significantly associated with the 1-year stroke recurrence risk in women may involve multi-level sex-specific pathophysiological interactions. First, hormone-mediated metabolic differences may play a crucial role: The decline in estrogen levels after menopause may weaken its protective effects on vascular endothelial function and insulin sensitivity, whereas an increase in TyG-BMI reflecting IR may further exacerbate endothelial dysfunction and atherosclerosis (32, 33). Additionally, although women tend to have a subcutaneous fat distribution, visceral fat accumulation (reflected by TyG-BMI) may more significantly drive the release of inflammatory factors such as IL-6 and TNF-α, thereby directly damaging vascular structures (34, 35). Second, sex differences in metabolic syndrome may amplify risks: Women are more sensitive to IR, especially in the context of hypertension. An increase in TyG-BMI, as a composite indicator of glucose and lipid metabolic disorders, may promote thrombosis and plaque instability through oxidative stress and chronic inflammation (36, 37). The synergistic effect of hypertension and elevated TyG-BMI may exacerbate vascular remodeling by activating the renin-angiotensin system and increasing sympathetic nervous system activity, and the thinner vascular walls in women are more susceptible to such damage (24). Third, sex-specific confounding by risk factors may contribute to these disparities. Stroke recurrence in men is predominantly driven by behavioral factors such as smoking and alcohol consumption (38), leading to a diluted association between TyG-BMI and stroke recurrence risk in men. Finally, sex-specific vascular pathophysiology may further explain this association: Women exhibit diminished cerebrovascular adaptability to blood pressure fluctuations, and elevated TyG-BMI may directly trigger ischemic events by exacerbating blood pressure variability or nocturnal hypertension (24, 39). Collectively, these mechanisms suggest that elevated TyG-BMI likely amplifies female-specific stroke recurrence risk through multiple sex-dimorphic pathways along the metabolic-inflammatory-vascular axis.

Several limitations need to be acknowledged in this study. First, the inability to track dynamic fluctuations in TyG-BMI levels may have influenced stroke outcomes during follow-up; subsequent research should incorporate serial TyG-BMI measurements to address this gap. Second, due to insufficient granularity regarding therapeutic interventions (mechanical thrombectomy, intravenous thrombolysis, or combined approaches), subgroup analyses for these cohorts were precluded. Third, exclusive recruitment from tertiary academic medical centers in Xi’an potentially introduced selection bias through underrepresentation of community hospital populations. Fourth, the classification of recurrent stroke subtypes (ischemic, transient, or hemorrhagic) was not available for all cases, primarily due to the limitations of telephone follow-ups where information was sometimes provided by family members. Additionally, the wide confidence intervals in the multivariate regression analysis may be due to the relatively small number of event cases. To mitigate these constraints, future studies will broaden recruitment criteria and increase sample size for more representative analyses.

In conclusion, elevated TyG-BMI is independently associated with a higher risk of 1-year stroke recurrence in patients with AIS and hypertension. Patients with TyG-BMI values in the Q3 and Q4 groups had a significantly higher 1-year stroke recurrence risk compared to those in the Q1 group. Furthermore, our study identified a statistically significant threshold effect for TyG-BMI at 231.45 in the overall AIS cohort. Below this threshold, each 1-unit increase in TyG-BMI was associated with a more pronounced increase in risk. Conversely, above 231.45, the impact on risk was substantially attenuated. However, subgroup analysis revealed a significant sex difference in the relationship between TyG-BMI and 1-year stroke recurrence risk. Specifically, among females with a TyG-BMI below 221.97, each unit increase in TyG-BMI was associated with a significantly greater increase in the risk of 1-year stroke recurrence compared to those above this threshold albeit no statistically significant threshold effect was observed in men. These findings necessitate sex-tailored metabolic management strategies and highlight TyG-BMI as a promising prognostic indicator. Clinicians should pay particular attention to these findings.

## Data Availability

The raw data supporting the conclusions of this article will be made available by the authors, without undue reservation.

## Supplementary material

Supplementary Figure 1 ROC curve analysis of the predictive value of TyG-BMI and its individual components for 1-year stroke recurrence in patients with AIS and with hypertension.
