# Supplementary material for: Elevated TyG-BMI significantly increases the 1-year stroke recurrence risk in patients with acute ischemic stroke and hypertension
Source: Front Endocrinol (Lausanne). 2025 Oct 22;16:1663393. doi: 10.3389/fendo.2025.1663393 (PMC12585975; doi:10.3389/fendo.2025.1663393)
Supplement: Supplementary Figure 1 — ROC curve analysis of the predictive value of TyG-BMI and its individual components for 1-year stroke recurrence in patients with AIS and with hypertension. [file DataSheet1.docx]

**Supplementary Table 1: Baseline clinical characteristics analysis of patients with AIS and hypertension, comparing those with and without 1-year stroke recurrence**

| Variables | Total (n = 1620) | Without stroke recurrence (n = 1531) | With stroke recurrence (n = 89) | P value |
| --- | --- | --- | --- | --- |
| Age, years | 65.2 ± 11.5 | 64.9 ± 11.5 | 70.8 ± 10.6 | < 0.001 |
| Gender |  |  |  | 0.466 |
| male | 978 (60.4) | 921 (60.2) | 57 (64) |  |
| female | 642 (39.6) | 610 (39.8) | 32 (36) |  |
| Educational level |  |  |  | 0.503 |
| elementary or below | 764 (47.2) | 717 (46.8) | 47 (52.8) |  |
| middle school | 333 (20.6) | 318 (20.8) | 15 (16.9) |  |
| high school or above | 523 (32.3) | 496 (32.4) | 27 (30.3) |  |
| Smoking, n (%) |  |  |  | 0.108 |
| Never smoking | 950 (58.6) | 898 (58.7) | 52 (58.4) |  |
| smoking cessation | 322 (19.9) | 298 (19.5) | 24 (27) |  |
| current smoking | 348 (21.5) | 335 (21.9) | 13 (14.6) |  |
| Drinking | 369 (22.8) | 355 (23.2) | 14 (15.7) | 0.103 |
| Time-to hospital admission | 42.4 ± 45.2 | 42.7 ± 45.4 | 37.7 ± 42.9 | 0.315 |
| BMI, kg/m2 | 24.1 ± 3.6 | 24.1 ± 3.6 | 24.4 ± 3.1 | 0.499 |
| SBP on admission (mmHg) | 150.4 ± 21.6 | 150.3 ± 21.5 | 152.5 ± 24.1 | 0.354 |
| DBP on admission (mmHg) | 87.6 ± 12.7 | 87.6 ± 12.6 | 88.2 ± 13.6 | 0.662 |
| Heart rate (times per minute) | 74.9 ± 10.5 | 74.6 ± 10.0 | 79.3 ± 16.4 | < 0.001 |
| NIHSS score on admission | 4.0 (2.0, 6.0) | 4.0 (2.0, 6.0) | 7.0 (3.0, 13.0) | < 0.001 |
| Walking within 48 hours of admission | 959 (64.0) | 935 (66) | 24 (29.6) | < 0.001 |
| Pneumonia, n (%) | 85 ( 5.2) | 63 (4.1) | 22 (24.7) | < 0.001 |
| Past medical history |  |  |  |  |
| Peripheral vascular history | 51 ( 3.1) | 45 (2.9) | 6 (6.7) | 0.057 |
| Prior stroke, n (%) | 512 (31.6) | 468 (30.6) | 44 (49.4) | < 0.001 |
| Diabetes mellitus, n (%) | 432 (26.7) | 405 (26.5) | 27 (30.3) | 0.421 |
| Atrial fibrillation, n (%) | 107 ( 6.6) | 90 (5.9) | 17 (19.1) | < 0.001 |
| Total cholesterol (mmol/L) | 4.4 ± 1.1 | 4.5 ± 1.1 | 4.4 ± 1.0 | 0.699 |
| Triglycerides (mmol/L) | 1.8 ± 1.5 | 1.8 ± 1.5 | 1.6 ± 1.1 | 0.429 |
| HDL-cholesterol (mmol/L) | 1.1 ± 0.3 | 1.1 ± 0.3 | 1.1 ± 0.3 | 0.883 |
| LDL-cholesterol (mmol/L) | 2.6 ± 0.9 | 2.6 ± 0.9 | 2.6 ± 0.8 | 0.807 |
| FPG (mg/dl) | 109.4 ± 43.5 | 108.7 ± 42.6 | 122.4 ± 55.4 | 0.004 |
| TyG | 8.8 ± 0.7 | 8.8 ± 0.7 | 8.9 ± 0.8 | 0.602 |
| TyG-BMI | 213.0 ± 37.8 | 212.8 ± 38.0 | 216.1 ± 35.2 | 0.431 |
| Aspartate aminotransferase (U/L) | 24.2 ± 21.8 | 24.1 ± 21.5 | 24.4 ± 26.0 | 0.919 |
| Alkaline phosphatase (U/L) | 24.8 ± 17.9 | 24.4 ± 15.7 | 31.6 ± 39.2 | < 0.001 |
| Alkaline phosphatase (U/L) | 79.6 ± 27.7 | 79.5 ± 27.8 | 81.0 ± 25.9 | 0.614 |
| Homocysteine (µmol/mL) | 21.7 ± 14.8 | 21.4 ± 14.5 | 26.8 ± 17.5 | 0.006 |
| Serum creatinine (µmol/L) | 77.1 ± 38.9 | 76.6 ± 39.4 | 84.5 ± 28.4 | 0.064 |
| eGFR (mL/min/1.73m2 ) | 74.7 ± 17.4 | 75.0 ± 17.4 | 69.0 ± 16.1 | 0.001 |
| Blood urea nitrogen (mmol/L) | 5.2 ± 1.9 | 5.2 ± 1.9 | 5.8 ± 2.2 | 0.004 |
| INR | 1.0 ± 0.2 | 1.0 ± 0.2 | 1.1 ± 0.2 | 0.299 |
| Uric acid (µmol/L) | 292.0 ± 98.2 | 292.7 ± 97.8 | 280.5 ± 104.9 | 0.265 |
| Leukocyte count (×109 /L) | 7.0 ± 2.5 | 6.9 ± 2.5 | 7.8 ± 2.6 | 0.002 |
| Platelet count (×109 /L) | 190.9 ± 60.3 | 191.2 ± 60.0 | 185.0 ± 64.5 | 0.35 |
| Antithrombotic drug therapy, n (%) | 1449 (89.4) | 1387 (90.6) | 62 (69.7) | < 0.001 |
| Antihypertensive drug therapy, n (%) | 1173 (72.4) | 1114 (72.8) | 59 (66.3) | 0.184 |
| Antidiabetic drug therapy, n (%) | 346 (21.4) | 328 (21.4) | 18 (20.2) | 0.788 |
| Lipid-lowering drug therapy, n (%) | 593 (36.6) | 563 (36.8) | 30 (33.7) | 0.559 |
| Anticoagulant drug therapy, n (%) | 35 ( 2.2) | 31 (2) | 4 (4.5) | 0.122 |

**Supplementary Table 2 Comparative analysis of clinical characteristics based on TyG-BMI > 231.45 and TyG-BMI < 231.45 groups.**

| Variables | Total (n = 1620) | TyG-BMI < 231.45 (n = 1208) | TyG-BMI ≥231.45 (n = 412) | P value |
| --- | --- | --- | --- | --- |
| Age, years | 65.2 ± 11.5 | 66.3 ± 11.4 | 61.9 ± 11.2 | < 0.001 |
| Gender |  |  |  | 0.086 |
| male | 978 (60.4) | 744 (61.6) | 234 (56.8) |  |
| female | 642 (39.6) | 464 (38.4) | 178 (43.2) |  |
| Educational level |  |  |  | 0.499 |
| elementary or below | 764 (47.2) | 580 (48) | 184 (44.7) |  |
| middle school | 333 (20.6) | 244 (20.2) | 89 (21.6) |  |
| high school or above | 523 (32.3) | 384 (31.8) | 139 (33.7) |  |
| Smoking, n (%) |  |  |  | 0.403 |
| Never smoking | 950 (58.6) | 705 (58.4) | 245 (59.5) |  |
| smoking cessation | 322 (19.9) | 249 (20.6) | 73 (17.7) |  |
| current smoking | 348 (21.5) | 254 (21) | 94 (22.8) |  |
| Drinking, n (%) | 369 (22.8) | 264 (21.9) | 105 (25.5) | 0.129 |
| Time-to hospital admission | 42.4 ± 45.2 | 42.1 ± 45.7 | 43.5 ± 44.0 | 0.588 |
| BMI, kg/m2 | 24.1 ± 3.6 | 22.9 ± 2.3 | 27.8 ± 3.9 | < 0.001 |
| SBP on admission (mmHg) | 150.4 ± 21.6 | 150.3 ± 21.9 | 150.8 ± 21.0 | 0.668 |
| DBP on admission (mmHg) | 87.6 ± 12.7 | 87.2 ± 12.5 | 88.9 ± 13.1 | 0.023 |
| Heart rate (times per minute) | 74.9 ± 10.5 | 74.3 ± 10.2 | 76.4 ± 11.0 | < 0.001 |
| NIHSS score on admission | 4.0 (2.0, 6.0) | 4.0 (2.0, 6.0) | 4.0 (2.0, 6.0) | 0.702 |
| Walking within 48 hours of admission, n (%) | 959 (64.0) | 708 (63.7) | 251 (65) | 0.632 |
| Pneumonia, n (%) | 85 ( 5.2) | 63 (5.2) | 22 (5.3) | 0.922 |
| Past medical history |  |  |  |  |
| Peripheral vascular history, n (%) | 51 ( 3.1) | 40 (3.3) | 11 (2.7) | 0.52 |
| prior.stroke, n (%) | 512 (31.6) | 390 (32.3) | 122 (29.6) | 0.314 |
| Diabetes mellitus, n (%) | 432 (26.7) | 256 (21.2) | 176 (42.7) | < 0.001 |
| Atrial fibrillation, n (%) | 107 ( 6.6) | 90 (7.5) | 17 (4.1) | 0.019 |
| Total cholesterol (mmol/L) | 4.4 ± 1.1 | 4.3 ± 1.0 | 4.8 ± 1.3 | < 0.001 |
| TG (mgldL) | 156.0 ± 131.9 | 126.6 ± 66.9 | 242.2 ± 213.0 | < 0.001 |
| HDL-cholesterol (mmol/L) | 1.1 ± 0.3 | 1.2 ± 0.3 | 1.1 ± 0.3 | < 0.001 |
| LDL-cholesterol (mmol/L) | 2.6 ± 0.9 | 2.6 ± 0.8 | 2.9 ± 1.0 | < 0.001 |
| FPG (mgldL) | 109.4 ± 43.5 | 101.7 ± 33.3 | 132.2 ± 59.2 | < 0.001 |
| TyG-BMI | 213.0 ± 37.8 | 196.8 ± 22.7 | 260.4 ± 33.1 | < 0.001 |
| Alanine aminotransferase (U/L) | 24.2 ± 21.8 | 23.0 ± 22.9 | 27.5 ± 17.6 | < 0.001 |
| Aspartate aminotransferase (U/L) | 24.8 ± 17.9 | 24.4 ± 17.2 | 25.9 ± 19.6 | 0.142 |
| Alkaline phosphatase (U/L) | 79.6 ± 27.7 | 79.6 ± 28.5 | 79.7 ± 25.4 | 0.944 |
| Homocysteine (µmol/mL) | 21.7 ± 14.8 | 22.0 ± 15.0 | 21.0 ± 14.1 | 0.324 |
| Serum creatinine (µmol/L) | 77.1 ± 38.9 | 77.4 ± 38.9 | 76.1 ± 39.0 | 0.564 |
| eGFR (mL/min/1.73m2 ) | 74.7 ± 17.4 | 74.0 ± 17.1 | 76.7 ± 18.0 | 0.007 |
| Blood urea nitrogen (mmol/L) | 5.2 ± 1.9 | 5.3 ± 2.0 | 5.1 ± 1.8 | 0.316 |
| INR | 1.0 ± 0.2 | 1.0 ± 0.2 | 1.0 ± 0.2 | 0.064 |
| Uric acid (µmol/L) | 292.0 ± 98.2 | 289.0 ± 95.5 | 300.7 ± 105.3 | 0.039 |
| Leukocyte count (×109 /L) | 7.0 ± 2.5 | 6.9 ± 2.4 | 7.3 ± 2.6 | 0.002 |
| Platelet count (×109 /L) | 190.9 ± 60.3 | 189.3 ± 60.4 | 195.7 ± 59.6 | 0.062 |
| Antithrombotic drug therapy, n (%) | 1449 (89.4) | 1083 (89.7) | 366 (88.8) | 0.641 |
| Antihypertensive drug therapy, n (%) | 1173 (72.4) | 860 (71.2) | 313 (76) | 0.061 |
| Antidiabetic drug therapy, n (%) | 346 (21.4) | 199 (16.5) | 147 (35.7) | < 0.001 |
| Lipid-lowering drug therapy, n (%) | 593 (36.6) | 380 (31.5) | 213 (51.7) | < 0.001 |
| Anticoagulant drug therapy, n (%) | 35 ( 2.2) | 29 (2.4) | 6 (1.5) | 0.255 |


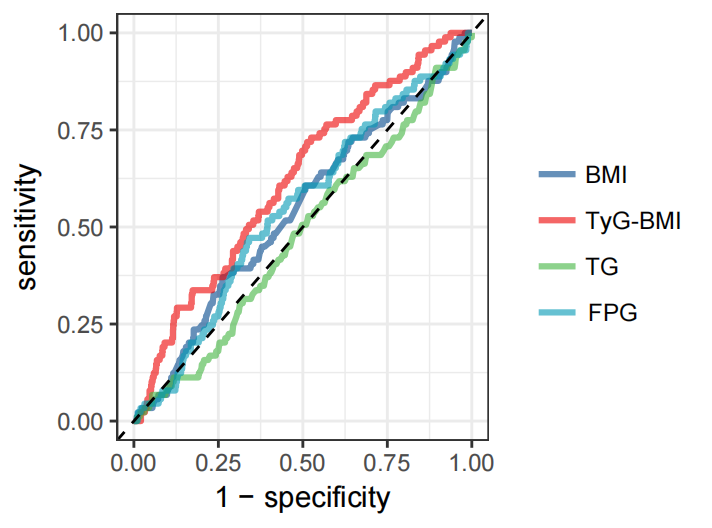


**Supplementary Figure 1: ROC curve analysis of the predictive value of TyG-BMI and its individual components for 1-year stroke recurrence in patients with AIS and with hypertension**
